# Advantages and limitations of microtiter biofilm assays in the model of antibiofilm activity of *Klebsiella* phage KP34 and its depolymerase

**DOI:** 10.1038/s41598-020-77198-5

**Published:** 2020-11-23

**Authors:** Agnieszka Latka, Zuzanna Drulis-Kawa

**Affiliations:** Department of Pathogen Biology and Immunology, Institute of Genetics and Microbiology, University of Wroclaw, ul. S. Przybyszewskiego 63, 51-148 Wrocław, Poland

**Keywords:** Biological techniques, Drug discovery, Microbiology

## Abstract

One of the potential antibiofilm strategies is to use lytic phages and phage-derived polysaccharide depolymerases. The idea is to uncover bacteria embedded in the biofilm matrix making them accessible and vulnerable to antibacterials and the immune system. Here we present the antibiofilm efficiency of lytic phage KP34 equipped with virion-associated capsule degrading enzyme (depolymerase) and its recombinant depolymerase KP34p57, depolymerase-non-bearing phage KP15, and ciprofloxacin, separately and in combination, using a multidrug-resistant *K. pneumoniae* biofilm model. The most effective antibiofilm agents were (1) phage KP34 alone or in combination with ciprofloxacin/phage KP15, and (2) depolymerase KP34p57 with phage KP15 and ciprofloxacin. Secondly, applying the commonly used biofilm microtiter assays: (1) colony count, (2) LIVE/DEAD BacLight Bacterial Viability Kit, and (3) crystal violet (CV) biofilm staining, we unravelled the main advantages and limitations of the above methods in antibiofilm testing. The diverse mode of action of selected antimicrobials strongly influenced obtained results, including a false positive enlargement of biofilm mass (CV staining) while applying polysaccharide degrading agents. We suggest that to get a proper picture of antimicrobials’ effectiveness, multiple examination methods should be used and the results must be read considering the principle of each technique and the antibacterial mechanism.

## Introduction

*Klebsiella pneumoniae* is a rod-shaped, nonmotile, encapsulated, Gram-negative bacillus prevalent in the environment as well as colonizing skin, mucosal tissues, and intestines as an inhabitant of the human microbiome^1^. World Health Organization (WHO) marked this dangerous opportunistic bacterium as a critical priority pathogen, resistant to all or nearly all available antibiotics^2,3^. *Klebsiella pneumoniae* is a representative of multidrug-resistant ESKAPE group (*Enterococcus faecium*, *Staphylococcus aureus*, *Klebsiella pneumoniae*, *Acinetobacter baumannii*, *Pseudomonas aeruginosa*, *Enterobacter* spp.) being able to infect the immune-compromised people as the nosocomial (hospital-acquired) pathogen, as well as colonize healthy individuals causing the community-acquired infections^4^.

*Klebsiella pneumoniae* capacity to cause infection and to evade the host immune system is assigned to the capsule (CPS), LPS, membrane transporters, and siderophores^5^. *Klebsiella pneumoniae* inhabiting both the environment and patients, is surrounded by a thick mucoid mainly acidic capsules, making a shield against desiccation, detergents, antibiotics and the host immune defense system^6^. *Klebsiella* efficiently forms a biofilm on medical devices like catheters or endotracheal tubes being the main source of nosocomial infections^4,7,8^. The wide usage of indwelling medical devices, such as respiratory support equipment and catheters, especially in neonatal wards and in long-term patients, is related to a high prevalence of *K. pneumoniae* infections^9,10^.

Biofilms are aggregates of bacterial cells embedded in a matrix composed of exopolysaccharides (EPS), extracellular DNA, and proteins^11^. The sessile cells are more resistant to antibiotics compared to planktonic cells^12,13^. The resistance can be physical due to biofilm matrix composition or physiological cell conditions depending on: (1) the metabolic state of bacteria versus antibiotic mode of action, (2) an adaptive tolerance (temporary, nongenetic tolerance due to antibiotic exposition), and (3) active resistance mechanisms such as antibiotic inactivation, antibiotic target modification or efflux pumps overproduction^14^. The nutrients limitation and hypoxia conditions in the biofilm center leading to slow cell metabolism thus decreasing the susceptibility to antibiotics. Since the biofilm resistance is based on complex mechanisms, the combination of multiple defensive strategies must be used to efficiently eradicate those bacterial structures.

One of the options is the application of natural bacterial viruses—phages, specifically recognizing and infecting a bacterial cell, propagating inside and causing cell lysis to release its phage progeny^15,16^. Phages do not easily diffuse within the biofilm matrix because of its viscosity, therefore to overcome this obstacle, some phages are equipped with virion-associated depolymerase, enzymes degrading extracellular polysaccharides (CPS, LPS, or EPS)^17^. Depolymerases are highly specific enzymes responsible for receptor recognition, binding, and degradation^18^. If not integrated into the virion mainly as tail spikes or tail fibers, depolymerase may occur also in a soluble form to digest polysaccharides helping progeny to escape the infected cell and diffuse freely in the biofilm matrix. Loosening and peeling off the biofilm structure, these phage enzymes can be facilitated as adjuvants providing an access to the sessile cells for other antimicrobials^19,20^.

In this work we utilized three different microtiter assays: (1) the colony count, (2), LIVE/DEAD BacLight Bacterial Viability method, and (3) crystal violet staining, to determine the antibiofilm effect of antimicrobials with a diverse mode of action. Using the multidrug-resistant *K. pneumoniae* model we wanted to present the discrepancies between different biofilm examination methods after the same way of biofilm treatment, unrevealing the advantages and limitations of commonly used assays^21,22^. We were testing the antibiofilm potential of *Klebsiella* lytic phage KP34, its recombinant KP34p57 capsule degrading depolymerase (non-bactericidal protein, possessing anti-virulent potential), lytic phage KP15 lacking virion-associated depolymerase, and ciprofloxacin (non-bacteriolytic antibiotic, acting on bacterial DNA gyrase) as a single agent and in the combination of multiple components.

## Results

In the anti-biofilm model, we used the multidrug-resistant *K. pneumoniae 77* strain producing extended-spectrum beta-lactamases (ESBL) combined with the resistance to aminoglycosides and trimethoprim/sulfamethoxazole. This strain remained susceptible to fluoroquinolones and carbapenems, therefore ciprofloxacin was selected as a model antibiotic for antibiofilm testing. The antibiofilm activity of lytic phage KP34 and its virion-associated depolymerase KP34p57, as a recombinant protein, was evaluated on *K. pneumoniae* 77 (K63) early (24 h), and maturated biofilms (48 h and 72 h). We wanted to verify if the recombinant KP34p57 (called later depolymerase) can effectively degrade the *K. pneumoniae* 77 biofilm matrix reducing its biomass and releasing bacterial cells embedded inside. The second task was to compare the antibiofilm efficiency of phage-based preparations versus ciprofloxacin, an antibiotic commonly used against multidrug-resistant *K. pneumoniae* strains. The third task was to test if the phage KP15 specific to bacterial protein receptor and non-infecting encapsulated *K. pneumoniae* 77 can eradicate the biofilm when mixed with phage KP34 or with recombinant KP34p57. Finally, we checked the most efficient antibiofilm composition of selected antibacterials to get a significant reduction of *K. pneumoniae* 77 mature biofilm. Following ways of treatment were examined with three different microtiter methods to compare their reliability in the biofilm eradiation monitoring.

### The untreated biofilm growth

**Figure 1:**
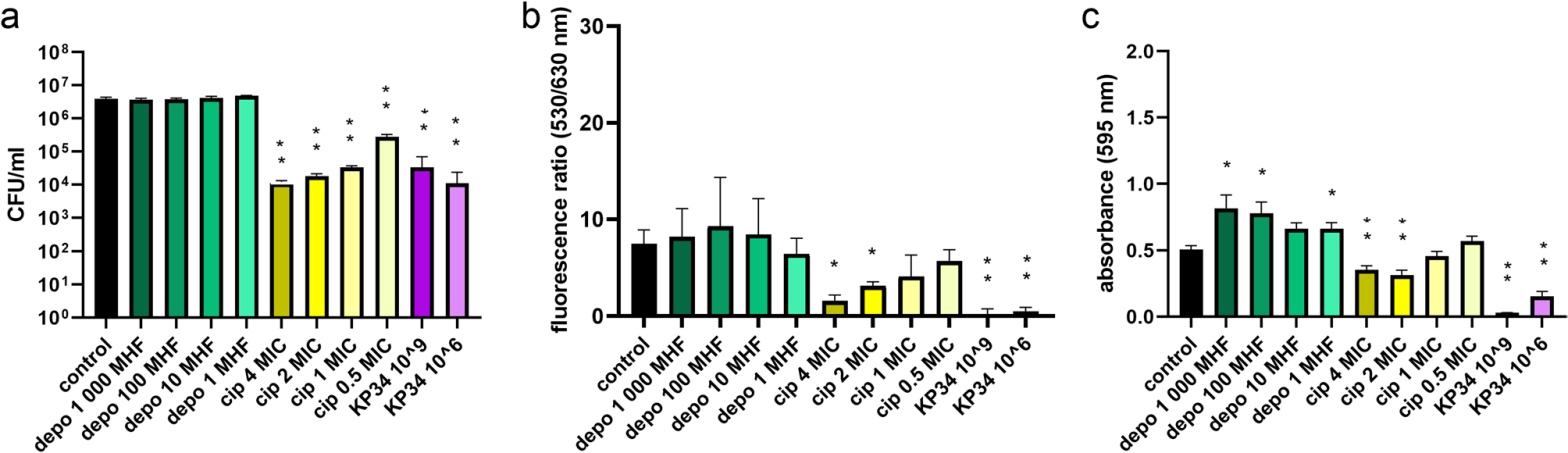
The influence of depolymerase, ciprofloxacin, and infective phage KP34 on *K. pneumoniae* 77 biofilm formed for 24 h on polystyrene pegs. One-day biofilm was treated for 2 h with: recombinant depolymerase KP34p57 originated from phage KP34 marked as depo, at concentrations corresponding to 1000 MHF (minimal halo forming concentration), 100 MHF, 10 MHF, and 1 MHF, green bars; ciprofloxacin marked as cip, at concentrations corresponding to 4 MIC (minimal inhibitory concentration), 2 MIC, 1 MIC, and 0.5 MIC, yellow bars; and lytic phage KP34 marked as KP34 (10^9^ PFU/ml and 10^6^ PFU/ml, violet bars). Biofilm eradication was examined with three different microtiter methods: (**a**) the colony count (CFU/ml); (**b**) LIVE/DEAD BacLight Bacterial Viability Kit with live to dead cells ratio at fluorescence 530/630 nm; (**c**) the crystal violet staining and the level of absorbance at 595 nm. *P-*value < 0.001 indicated as two asterisks, and *P*-value between 0.01 and 0.001 indicated as one asterisk, were considered statistically significant.

**Figure 2:**
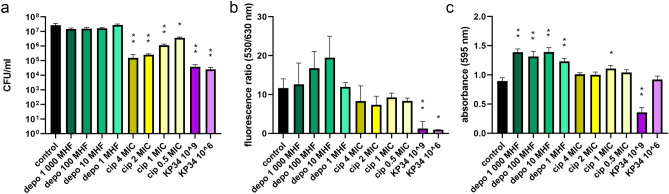
The influence of depolymerase, ciprofloxacin, and infective phage KP34 on *K. pneumoniae* 77 biofilm formed for 48 h on polystyrene pegs. Two-day biofilm was treated for 2 h with: recombinant depolymerase KP34p57 originated from phage KP34 marked as depo, at concentrations corresponding to 1000 MHF (minimal halo forming concentration), 100 MHF, 10 MHF, and 1 MHF, green bars; ciprofloxacin marked as cip, at concentrations corresponding to 4 MIC (minimal inhibitory concentration), 2 MIC, 1 MIC, and 0.5 MIC, yellow bars; and lytic phage KP34 marked as KP34 (10^9^ PFU/ml and 10^6^PFU/ml, violet bars). Biofilm eradication was examined with three different microtiter methods: (**a**) the colony count (CFU/ml); (**b**) LIVE/DEAD BacLight Bacterial Viability Kit with live to dead cells ratio at fluorescence 530/630 nm; (**c**) the crystal violet staining and the level of absorbance at 595 nm. *P*-value < 0.001 (indicated as two asterisks) and *P-*value between 0.01 and 0.001 (indicated as one asterisk) were considered statistically significant.

**Figure 3:**
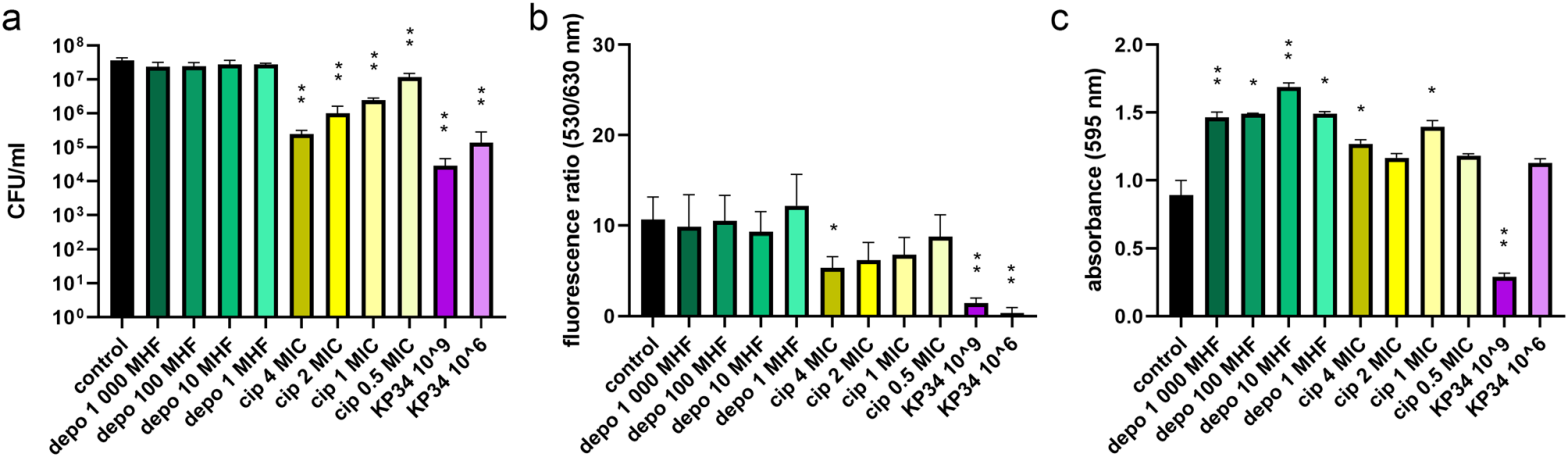
The influence of depolymerase, ciprofloxacin, and infective phage KP34 on *K. pneumoniae* 77 biofilm formed for 72 h on polystyrene pegs. Three**-**day biofilm was treated for 2 h with: recombinant depolymerase KP34p57 originated from phage KP34 marked as depo, at concentrations corresponding to 1000 MHF (minimal halo forming concentration), 100 MHF, 10 MHF, and 1 MHF, green bars; ciprofloxacin marked as cip, at concentrations corresponding to 4 MIC (minimal inhibitory concentration), 2 MIC, 1 MIC, and 0.5 MIC, yellow bars; and lytic phage KP34 marked as KP34 (10^9^ PFU/ml and 10^6^ PFU/ml, violet bars). Biofilm eradication was examined with three different microtiter methods: (**a**) the colony count (CFU/ml); (**b**) LIVE/DEAD BacLight Bacterial Viability Kit with live to dead cells ratio at fluorescence 530/630 nm; (**c**) the crystal violet staining and the level of absorbance at 595 nm. *P*-value < 0.001 (indicated as two asterisks) and *P-*value between 0.01 and 0.001 (indicated as one asterisk) were considered statistically significant.

Bacteria embedded in the biofilm structure (control samples) formed for 24 h reached 6 logs of CFU/ml (Fig. 1a, black bar) and ten times higher level of cells (10^7^) after two and three days of development (Fig. 2a and 3a, black bars). The ratio of living/dead bacteria after the first 24 h achieved a value of 7.5 and further rose to the level between 10.6 and 11.7 (Figs. 1, 2, 3b, black bars). Similarly, the CV staining showed a gradual increase in biomass during a prolonged biofilm formation and stabilized after 48 h (Figs. 1, 2, 3c, black bars).

### The antibiofilm activity of phage KP34

At the beginning of the study, an antibiofilm activity was determined for each agent separately at the panel of different concentrations or titer (Figs. 1, 2, 3). Lytic phage KP34 activity was tested for two titers (10^9^ PFU/ml and 10^6^ PFU/ml). The colony count method and live/dead cell kit were recognized as the most effective methods in biofilm eradication monitoring while treated with the phage. It turned out that both phage titers were able to significantly reduce the colony count in the range of 2–3 logs, with the highest reduction (3.1 logs) achieved for 72 h biofilm treated with 10^9^ PFU/ml (Figs. 1, 2, 3a, violet bars). Similarly, the live/dead cell ratio was statistically significant reduced (*P*-value < 0.001, or 0.01–0.001) from 86 to 99.7% for all stages of biofilm formation (Figs. 1, 2, 3b, violet bars). Bacterial cell lysis caused by phage infection led also to biofilm structure exfoliation, while the lysed cells released their DNA ready to be bound with propidium iodide. It means less number of living cells and more free DNA released from dead cells were responsible for a significant drop in the live/dead ratio. The crystal violet visualized the biofilm biomass reduction of 94%, 60%, and 67% for 24 h, 48 h, and 72 h biofilm, respectively, when a high phage titer was applied (Figs. 1, 2, 3c, violet bars). A lower phage titer (10^6^ PFU/ml) reduced only the 24 h biofilm (71%), whereas no statistically significant influence against older biofilms was seen. Probably the level of phage propagation was too low to cause biofilm exfoliation and partially degraded biomass (negatively charged) interacted with a high number of crystal violet molecules. For further investigation of combined antibiofilm therapy the in-between 10^8^ PFU/ml phage titer was chosen to observe a possible improved effect of antibacterials combination.

### The antibiofilm activity of phage depolymerase

First, the degrading activity of the prepared recombinant enzyme was verified in the serial dilution on a bacterial lawn (spot test), and the extracted *K. pneumoniae* 77 capsular polysaccharides by zymography assay (Supplementary Materials, Figure S2a, and S1b, respectively). In antibiofilm experiments, four concentrations of KP34p57 depolymerase were tested: 7.5 ng/ml (corresponding to the minimal concentration of depolymerase able to form transparent halo zone, called further MHF—minimal halo forming concentration), 75 ng/ml (10 MHF), 750 ng/ml (100 MHF) and 7500 ng/ml (1000 MHF). None of the tested concentrations gave significant changes in the colony count after 2 h of incubation with the enzyme regardless of the biofilm age (Figs. 1, 2, 3a, green bars). Indeed, the degradation of the bacterial capsule does not lead to cell death. It turned out that 2-h incubation was not enough to release the sessile cells from the biofilm matrix. The application of LIVE/DEAD BacLight Bacterial Viability Kit (Figs. 1, 2, 3b green bars) confirmed that the treatment of 24 h biofilm with 1 MHF and 72 h biofilm with 10 MHF concentrations led only to a slight, statistically non-significant live/death ration reduction (14% and 12%, respectively). Depolymerase treatment was not expected to influence the live/dead cells ratio, since it does not cause the cell death or cell membrane damage. These results were consistent with the CFU count method.

On the contrary, the crystal violet staining indicated the biofilm biomass increase after the application of each KP34p57 depolymerase concentration (Figs. 1, 2, 3c, green bars), with the highest range of 164–190% compared to the control sample. The changes induced by depolymerase treatment of 48 h- and 72 h biofilm were statistically significant (*P* < 0.01).

Since there was no significant influence of any tested enzyme concentration on the colony count and live/dead cells ratio we decided to choose the 10 MHF for further investigation. This concentration had the highest impact on 72 h- old biofilm (visualized by CV staining) and on the live/dead ratio decrease (however statistically non-significant).

### The antibiofilm activity of ciprofloxacin

A bactericidal effect of ciprofloxacin was tested at MICs concentrations established for planktonic *K. pneumoniae* 77 cells: 4 MIC = 1 µg/ml, 2 MIC = 0.5 µg/ml, 1 MIC = 0.25 µg/ml and 0.5 MIC = 0.125 µg/ml. Each tested concentration resulted in a statistically significant decrease of the colony count after two-hour treatment (Figs. 1, 2, 3a, yellowish bars). The lowest influence was observed for the mature biofilm treated with 0.5 MIC (0.5 log and 0.9 log reduction for 72 h and 48 h biofilm, respectively) and 1.2 log reduction of cells embedded in the early biofilm. An increased antibiotic concentration (1, 2, and 4 MICs) were more efficient reducing in the colony count between 1 and 2.6 logs (Figs. 1, 2, 3a, yellowish bars).

The SYTO 9/propidium iodide staining indicated 79%, 29%, and 50% reduction of live/dead cells ratio starting from an early to mature biofilm after treatment with 4 MIC of ciprofloxacin (Figs. 1, 2, 3b, yellowish bars). The influence on the early and mature biofilm was statistically significant (*P-*value between 0.01 and 0.001), while a 48 h biofilm was not harmed. Lower antibiotic concentrations (0.5–2 MICs) caused non-significant changes (18–45%). Only 2 MIC of ciprofloxacin significantly reduced (58%) the live/dead ration of cells embedded in early biofilm. Ciprofloxacin concentration of 4 MIC was chosen for further investigation, as it was effective on the mature biofilm. Live/dead ratio results were not consistent with the data obtained for the colony count method, where each antibiotic concentration had a significant impact on the CFU number. These differences may be based on the ciprofloxacin mode of action, which led to bacterial cell death but not to bacterial cell lysis. In this case, the dead cells are probably not efficiently penetrated by propidium iodide, thus the reduction of live/dead cells ratio was not observed. To confirm that hypothesis, the additional experiment was performed on planktonic cells treated accordingly for 2 h with 4 MIC of ciprofloxacin and stained with the LIVE/DEAD BacLight Bacterial Viability Kit (Supplementary materials, Fig. S4). The fluorescent microscopy analysis showed the reduced number of living cells compared to untreated control and the elongated shape of bacterial cells after DNA replication blocking caused by ciprofloxacin. Treated cells were both stained with green and red dye but the morphologically changed cells were around 10 times bigger/longer than untreated ones, which may explain the differences in the colony count versus the live/dead ratio.

The CV staining indicated the biofilm eradication only for early biofilm treatment with 4 MIC, 2 MIC, and 1 MIC of ciprofloxacin (31%, 39%, and 10% of biomass reduction, respectively), and only the changes for 4 MIC and 2 MIC were recognized as statistically significant (*P*-value < 0.001). The mature biofilm seemed to be stimulated by antibiotic treatment, having a statistically significant increase of 23% (1 MIC on 48 h biofilm), as well as of 43% for 4 MIC and 57% for 1 MIC against 72 h biofilm (Figs. 1, 2, 3c, yellowish bars). For further antibiofilm experiments of combined therapy, only the 4 MIC ciprofloxacin concentration was chosen.

### The antibiofilm activity of combined preparations

**Figure 4:**
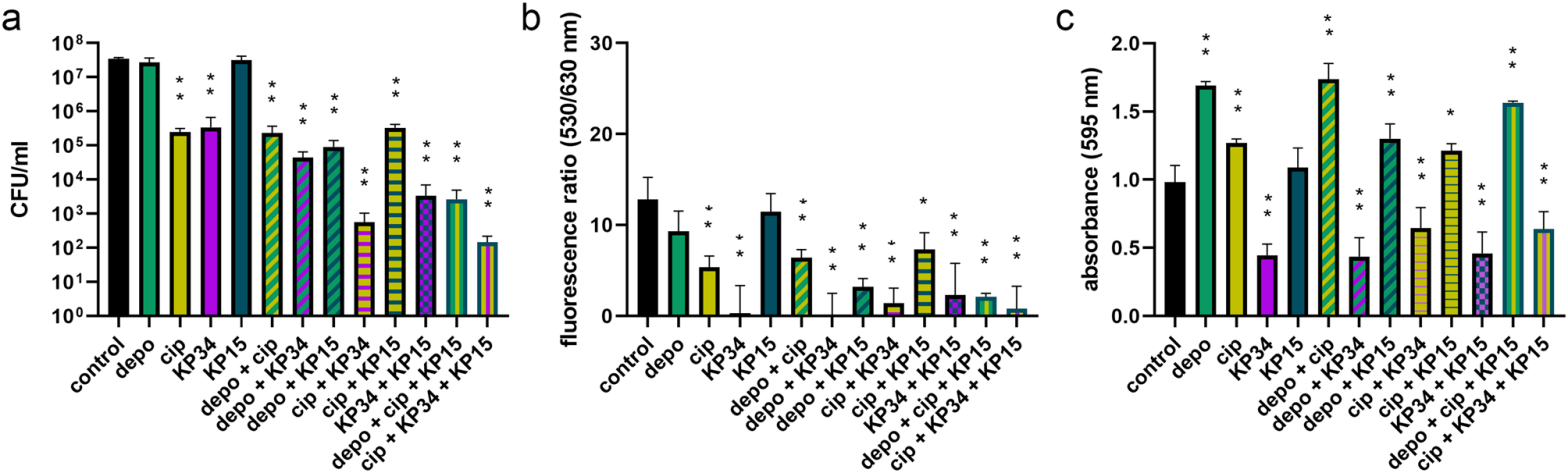
The antibiofilm activity of combined preparations composed of KP34p57 recombinant depolymerase (depo) at concentration corresponding to 10 MHF (minimal halo forming concentration), ciprofloxacin (cip) at concentration corresponding to 4 MIC (minimal inhibitory concentration), lytic phage KP34 specific to *K. pneumoniae* 77 (KP34, 10^8^ PFU/ml), and lytic phage KP15 non-specific to *K. pneumoniae* 77 (KP15, 10^8^ PFU/ml) on *K. pneumoniae* 77 biofilm formed for 72 h on polystyrene pegs. The 72 h biofilm was treated for two hours with different combinations of antimicrobials as indicated under the columns. Plus ( +) between abrreviations indicates that two or three antimicrobials were mixed and used simultaneously. Biofilm eradication was examined with three different microtiter methods: (**a**) the colony count (CFU/ml); (**b**) LIVE/DEAD BacLight Bacterial Viability Kit with live to dead cells ratio at fluorescence 530/630 nm; (**c**) the crystal violet staining and the level of absorbance at 595 nm. *P*-value < 0.001 (indicated as two asterisks) and *P-*value between 0.01 and 0.001 (indicated as one asterisk) were considered statistically significant.

The colony count in the biofilm formed for 48 h and 72 h stayed at the same level, therefore only 72 h mature biofilm was selected for further antibiofilm experiments. For combined antibiofilm treatment the following antimicrobials were used: 10^8^ PFU/ml of phages, 10 MHF of KP34p57 depolymerase, and 4 MIC of ciprofloxacin (Fig. 4).

Depolymerase did not improve nor negatively influenced antibiotic activity, since the combination of both agents (depolymerase plus ciprofloxacin) and ciprofloxacin itself made the same impact on biofilm (2.2 logs of CFU reduction and 50% in live/dead ratio, *P*-value < 0.001, Fig. 4a,b, green/yellowish striped and yellowish bars). On the contrary, CV staining indicated again an increase of biofilm biomass for depolymerase/antibiotic combination and depolymerase treatment itself (~ 170% compared to the control, *P*-value < 0.001, Fig. 4c).

No significant differences were observed after biofilm treatment with depolymerase and depolymerase-producing phage KP34 (Fig. 4, green/violet striped and violet bars). In contrast, enzyme combined with phage KP15 allows for phage infection with 2.6 logs reduction in CFU and 75% in live/dead ratio (*P*-value < 0.001, Fig. 4 green/navy blue striped). Depolymerase enabled phage KP15 receptor exposition and followed infection since the phage KP15 receptor is hidden beneath the bacterial capsule. A similar effect was observed when phage KP15 was co-infected with depolymerase-bearing phage KP34.

The most effective antibiofilm combinations were: (1) ciprofloxacin with phage KP34, (2) depolymerase producing phage KP34 together with depolymerase-non-bearing phage KP15, and (3) both phages mixed with an antibiotic. For the aforementioned combinations the biofilm eradication was detected at the level of 4–5.7 logs reduction in the colony count, 82–94% drop of the live/dead ratio, and between 34 and 53% biomass decrease, (*P-*value < 0.001, Fig. 4). Biofilm treatment with a triple cocktail containing depolymerase, depolymerase-non-bearing phage KP15, and ciprofloxacin resulted in a high CFU and live to dead ratio reduction as well (4.1 logs and 83.5% respectively, *P*-value < 0.001, Fig. 4a,b, green/yellowish/navy blue vertical striped bar). The CV staining method indicated a significant biomass increase for most of the depolymerase application samples.

## Discussion

In this study, we have two main aims. The first was to determine the activity of a recombinant phage-encoded enzyme (depolymerase KP34p57), lytic phages (producing and non-producing depolymerase), and commonly used antibiotic (ciprofloxacin) in the matter of multidrug-resistant *K. pneumoniae* biofilm eradication. The second aim was to use the above model to unravel the most common problems regarding inconsistent results obtained in the microtiter antibiofilm assays when determining the activity of different agents including antibiotics, phages, and matrix-degrading enzymes.

### The eradication efficiency of *K. pneumoniae* biofilm by different agents

*Klebsiella pneumoniae* is well protected from chemicals and the host immune system by a thick polysaccharide capsule, and transferring to sessile cells embedded in the biofilm matrix. Phages equipped with depolymerase can use these specific enzymes to degrade matrix structure and gain access to bacteria hidden inside^17^. It was suggested that depending on biofilm topography, phages could travel within through the channels and pores causing further structure exfoliation^17^. Therefore, recombinant depolymerases and phage equipped with virion-associated depolymerase are interesting candidates as antibiofilm agents alone or in combination with other antimicrobials. The antibiofilm potential of phage-borne enzymes was already confirmed in the literature for phage SH-KP152226—encoded depolymerase degrading *K. pneumoniae* biofilm^23^. The biofilm was treated with Dep42 depolymerase at concentrations of 1 or 10 µg/ml reducing the CFU of 0.3 logs. When the biofilm examination was performed with CV staining authors observed 0.4–0.7 of absorbance reduction. In another study, an enzyme derived from phage KPO1K2 (not recombinant) was used to treat *K. pneumoniae* B5055 biofilm decreasing the colony count of 3.3 logs^24^. The followed study verified the antibiofilm activity of the aforementioned phage KPO1K2 depolymerase and bacteria-borne (*Aeromonas punctate*) polysaccharide degrading enzyme against *K. pneumoniae* B5055 sessile cells. It turned out that bacterial enzyme was slightly more effectively giving an average reduction in the bacterial count of 3.3 logs compared to 2.2 logs observed after phage depolymerase treatment^25^. In our experiments, contrary to the above studies, recombinant depolymerase KP34p57 did not significantly reduce the number of colony count or live/dead cells ratio when applied alone at any concentration for two-hour treatment. Only 72 h biofilm treated with 75 ng/ml depolymerase was degraded of around 12–24% (according to live/dead ratio and colony count, respectively) what was still statistically insignificant. Published results were also contradictory to our crystal violet staining, where we saw a significant increase of the biomass for all tested enzyme concentrations and biofilm age. These discrepancies might be explained by the differences in the degrading activity of applied enzymes and the duration of treatment. Our phage-borne depolymerase KP34p57 could be not enough effective within 2 h to disrupt the biofilm matrix and release most of the embedded cells contrary to Dep42 or *Aeromonas punctate* depolymerases^25^. The low efficiency of the enzyme might also be addressed to the differences in the exopolysaccharide composition of the matrix, and CPS^17^. However, we have proved that the combination of depolymerase KP34p57 and phage KP15 was very effective in biofilm eradication reducing the CFU of 2.6 logs and 75% of live/dead ratio. Therefore, we might conclude that phage enzymes are promising adjuvants to non-depolymerase-producing phages.

In this study, we also tested the activity of ciprofloxacin for *Klebsiella* biofilm eradication. Antibiotic treatment led to a maximum of 2 logs reduction in the colony count both for early and mature biofilms, however, the efficiency was decreasing along with biofilm age. One of the reasons might be the limited diffusion of antibiotics within the mature matrix, therefore a higher concentration of ciprofloxacin was needed to successful penetration and killing of sessile cells (4 MIC). A similar phenomenon was reported for *P. aeruginosa* biofilm where ciprofloxacin diffusion within biofilm was reduced due to the drug-matrix components binding^26^. The second reason can be associated with the spatial variation in the physiological feature since bacteria in a slowly growing state are less susceptible to the antibiotic action^12^. That was confirmed for *Klebsiella* strains biofilm eradication with 10 MIC ciprofloxacin used in another study^12^. Considering the unexpected increase in biofilm mass values detected by CV staining we might suggest that the limited ciprofloxacin penetration led to the impact of subinhibitory concentration of antibiotics in the nearhood of cells, forcing bacterial cells to increased EPS production. That effect was previously reported for a sub-MIC ciprofloxacin concentration applied against *P. aeruginosa* and *E. coli* resulting in the enhanced matrix synthesis and biofilm formation^27^. Another explanation could be based on the release of cell components to the biofilm matrix increasing the total level of negatively charged moieties reacting with crystal violet molecules.

In the presented study, the depolymerase did not improve ciprofloxacin activity giving the level of eradication corresponding to antibiotic action itself. Verma and co-workers showed also ciprofloxacin/depolymerase combination treatment of 5 days—old *K. pneumoniae* biofilm, but the improvement was only significant when depolymerase pretreatment was followed by ciprofloxacin application^24^. A similar effect was reported in another study^23^ describing a slightly higher eradication ability of phage enzyme Dep42/polymyxin (6 MIC) cocktail against *K. pneumoniae*. It can rather be concluded that phage depolymerases do not interrupt the activity of antibiotics. In contrast, the combination of gentamicin/phage/phage depolymerase was very effective in the treatment of *K. pneumoniae* B5055 biofilm^25^. While gentamicin itself did not influence the colony count, the aforementioned mixture with phage and depolymerase led to 4 and 5 logs reduction, respectively, but the older biofilm was less sensitive to the treatment procedure^25^.

Regarding phage-borne capsule depolymerase activity in *K. pneumoniae* biofilm eradication, we may conclude that it depends on the biofilm-forming abilities of strain, variations in matrix composition as well as dissimilarity in enzymatic efficiency of depolymerase.

The best antibiofilm results were obtained here where lytic phage KP34 was applied, and all three applied monitoring methods were consistently indicating a mature biofilm eradication: 3 logs reduction in the colony count (~ 99.9%), 86% and 67% reduction of live/dead ratio, and biomass, respectively. Analogous results were reported for lytic phage TSK1 infecting *K. pneumoniae* biofilm affecting both a high biomass reduction (> 95%) and bacterial load removal up to 90–100%^28^. The efficiency of phage infection is a decisive factor in biofilm eradication, however, the susceptibility of sessile cells to phage infection is strain- and biofilm age-dependent. The supplementation of lytic phage KP34 with ciprofloxacin significantly improved antibiofilm activity from 2 logs to almost 5 logs of CFU reduction in the mature biofilm, in our study. On the contrary, the combination of ciprofloxacin with phage KPO1K2 had no improving effect^29^. The authors, however, reported that a drug/phage treatment resulted in a decreased rate of antibiotic and phage resistance emergence^29^. The following studies on older biofilms, conducted by this group, revealed that ciprofloxacin itself can reduce the colony count of 2 logs but only up to 96 h biofilm. The 5–7 days biofilms were not susceptible to antibiotic treatment, whereas were still susceptible to phage infection resulting in 3 logs of CFU reduction^24^. Verma and co-workers compared also the activity of two bacteriophages: producing and non-producing depolymerase but exhibiting a similar potential in planktonic *K. pneumoniae* cell infection. Phage non-producing depolymerase, in contrary to depolymerase-producer, was not able to reduce the sessile colony count^24^. Here we combined KP34 and KP15 phages (a depolymerase producing and non-producing, respectively), achieving a high antibiofilm effect with 4 logs reduction in the colony count. Phage KP34 itself caused 2 log-decrease whereas KP15 alone was not able to propagate at all. The phage cocktail activity was further improved by the addition of ciprofloxacin (reaching 5.7 logs reduction). The fact that the simultaneous delivery of depolymerase, antibiotic, and phages does not negatively influence the activity of each compound is highly promising from the therapy point of view. The combination of multiple antimicrobial compounds, apart from improved antibiofilm effect, might also be useful in limiting the resistance emergence^29^.

### The advantages and limitations of different biofilm monitoring assays

**Figure 5:**
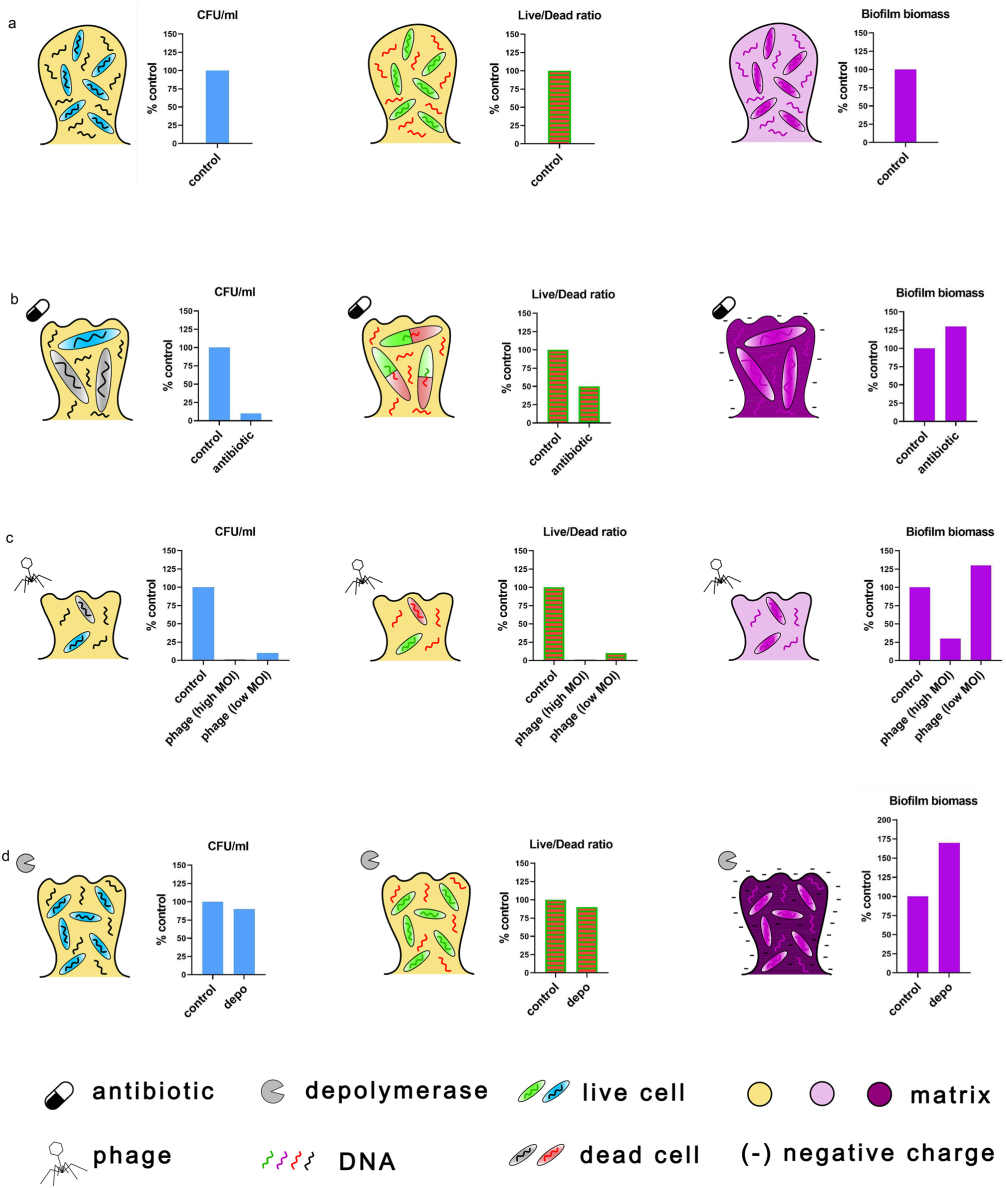
The advantages and limitations of different biofilm monitoring assays used in this study: the colony count (left), LIVE/DEAD BacLight Bacterial Viability Kit (middle), biomass staining with crystal violet (right). The antibiofilm strategies were tested for: (**a**) untreated biofilm; (**b**) antibiotic application; (**c**) phage infection at a high and low MOI; (**d**) depolymerase action. The colony count indicates only culturable bacteria, thus the antibiotic and phage application showed the best antibiofilm effect. The life/dead ratio gave inconsistent results for ciprofloxacin treatment because of the specific antibiotic mode of action causing the elongation of cells and dyes accumulation. The CV staining is based on the dye interactions with the negative charge thus showed a false enlargement of biofilm after antibiotic, depolymerase, and phage at a low MOI application.

The second aim of the study presented here was to determine and compare the usefulness of different microtiter techniques applied commonly for antibiofilm monitoring. Therefore we used three different assays allowing us to evaluate various biofilm features. The first one was the colony count, the second was the live/dead ratio determining method, and the last was CV staining (Fig. 5).

Each method has its pros and cons and focuses on different aspects of complex biofilm structure^22^. The colony count reveals only culturable bacteria meaning that the subpopulation of viable but nonculturable bacteria is not detected^30,31^. What is more, the cell count can be easily underestimated due to the improper detachment of biofilm structure and bacterial cell aggregation. As presented in Fig. 5, the strongest CFU reduction was shown for drug and phage treatment (Fig. 5b,c), but only for the later one, it was also accompanied by biomass reduction.

The LIVE/DEAD BacLight Bacterial Viability Kit, with propidium iodide as a compound^32,33^, has also limitations related to the antimicrobial’s mode of action. The dye which is supposed to indicate dead cells can only penetrate and stain DNA when the bacterial cell membrane is damaged. Bacteria not being able to reproduce in a suitable environment should be marked as “dead” according to the common viability criterium, but if the cell membrane stays impermeable it will be scored as “alive” when stained with propidium iodide. In our case, the mature biofilm treated with ciprofloxacin was reduced by 2 logs in the colony count, thus one could expect that a live/dead ratio reduction should be around 99%, while in fact, it was only 50% (Fig. 5b). Ciprofloxacin, a bactericidal antibiotic belonging to fluoroquinolones, acts on topoisomerase IV and DNA gyrase blocking the replication process and cell division^34^. Theoretically, dead microorganisms lose their capability to maintain their membranes intact, which should enable the penetration of propidium iodide (a component of LIVE/DEAD BacLight Bacterial Viability Kit) into the cell. Our study revealed that the inconsistent results in CFU count versus staining came from the formation of elongated cells halted in the division process by ciprofloxacin, thus the life/dead ratio remained relatively high compared to untreated culture.

Even if the bacterial cells are killed, the biofilm matrix may not be removed, becoming the starting point for biofilm regrowth, thus the third method we used was crystal violet (CV) staining. The CV as a positively charged stain binds to the negatively charged components like polysaccharides, proteins, or nucleic acids. Any antimicrobial agent causing the increase of negatively charged residues will also enhance the formation of CV complex giving to a false interpretation of biofilm biomass enlargement. In this study, the biomass staining showed the eradication effect only when lytic phage was applied at high MOI (Fig. 5c), whereas in the remaining cases, the biomass seemed to increase. It might be due to the negatively charged polysaccharides overproduction (i.e. as a response to the antibiotic treatment, Fig. 5b) or due to an increase of negatively charged components released from the dead cells and trapped in the matrix, or due to the effect of EPS/CPS enzymatic degradation by phage-borne depolymerase (Fig. 5d). Other studies proved the efficiency of depolymerase treatment with crystal violet staining^23,35^, which was opposite to results from our study, where we observed an extensive biomass increase after depolymerase treatment. It must be also stressed that the differences in biomass level might also be due to: (1) discrepancies in biofilm formation rate, (2) biofilm structure and matrix composition, and (3) degrading enzyme efficiency. Moreover, the variability in CV staining results may be due to technical protocol. Also, the methods of biofilm development itself differ among the studies: (1) can be grown for a different period, (2) developed in the wells or on pegs, (3) starting CFU/ml may differ, or (4) the growing medium can be exchanged/refreshed or not. It seems that CV staining is a convenient method for biofilm growth monitoring where no antimicrobials are applied, but for antibiofilm tests, this technique must be carefully interpreted taking into account the basis of CV interactions.

Depending on the chosen methodology for antibiofilm analysis, the results of antimicrobial activity might differ significantly, see Fig. 5, thus it is essential to know the mode of action of the applied agent. To properly analyze the effectiveness of the antimicrobial, the multiple examination microtiter methods should be applied and the results must be verified considering the biological and chemical background of each technique.

## Summary

In conclusion, the most effective antibiofilm combinations were: (1) ciprofloxacin with depolymerase producing phage KP34, (2) depolymerase producing phage KP34 together with depolymerase-non-bearing phage KP15, and (3) both phages mixed with the antibiotic. A triple cocktail containing depolymerase, depolymerase-non-bearing phage KP15, and ciprofloxacin resulted in a high CFU and live/dead ratio reduction as well. Lytic activity of phage KP34 itself and in combination with other antimicrobials possess a satisfactory antibiofilm potential. Depolymerase originated from phage KP34 can be used as a supportive antibiofilm agent for depolymerase-non-bearing phages.

Results acquired from different microtiter biofilm assays may differ depending on the mode of action of a particular antimicrobial. Colony count can be underestimated due to bacterial aggregation or detachment of biofilm structure. Live to dead cells ratio calculated from LIVE/DEAD BacLight Bacterial Viability Kit staining strongly depends on the bacterial membrane integrity. Biofilm biomass monitored with crystal violet can be easily overestimated by the variation in negatively charged components. Thus overall the biofilm eradication assays should be carefully interpreted.

## Materials and methods

### Phage propagation

Phages KP34 and KP15 were propagated on the respective hosts being *Klebsiella pneumoniae* 77 and *K. pneumoniae* ATCC 700603 for phage KP34 and KP15, respectively. *Klebsiella pneumoniae* 77 from the collection of the Institute of Genetics and Microbiology, University of Wroclaw, Poland, is a multidrug-resistant strain, producing extended-spectrum beta-lactamases (ESBL) combined with the resistance to aminoglycosides and trimethoprim/sulfamethoxazole. This strain remains susceptible to ciprofloxacin and carbapenems, therefore ciprofloxacin was chosen for antibiofilm testing in this study. The phage lysate at MOI of 0.1–0.01 was added to 10 ml of a logarithmically growing bacterial culture in Tryptic Soy Broth (bioMerieux, Marcy l'Etoile, France). The incubation at 37 °C with the agitation was performed for 3 h. The phage lysate was mixed with 0.5 ml of chloroform (VWR, Radnor, Pennsylvania, USA), the top phase was separated and spun down (5000 × *g*, 30 min, 4 °C). The supernatant was subsequently sterilized by filtration (0.22 μm, Merck Millipore, Burlington, Massachusetts, USA). The phage titer was established using the double-agar layer method^36^.

### Depolymerase cloning and production

The KP34p57 depolymerase specific to the K63 capsular serotype (YP_003347651.1, locus tag KP34p57) was found to be homologous to pectin lyase (confidence 68,7%; id 18%; aa 32-371) by PHYRE2 online tool^37,38^. *KP34p57* gene was amplified by PCR using Pfu DNA polymerase (Thermo Fisher Scientific, Waltham, Massachusetts, USA) and the specific primers (F: ATGGCACTCACTAAACTAGTAGATGCAGG and R: ACCAGTGAGTTCAGATGGAGCAAAGCAGC) and followed by post-amplification 3′ A-overhang addition by DreamTaq polymerase (Thermo Fisher Scientific, Waltham, Massachusetts, USA). Subsequently, the amplicons were cloned into the pEXP5-CT/TOPO vector (Invitrogen, Thermo Fisher Scientific, Waltham, Massachusetts, USA) with a C-terminal histidine tag according to the manufacturer's conditions. Transformation of chemocompetent *E. coli* TOP10 (Invitrogen, Waltham, Massachusetts, USA) for plasmid propagation and isolation was followed by Sanger sequencing (Genomed, Warsaw, Poland) of constructs that were verified by colony PCR as possessing proper construct. Correct constructs were used for *E. coli* BL21(DE3) transformation and subsequent protein expression.

*Escherichia coli* BL21(DE3) cells were grown in Luria Broth (Biomaxima, Lublin, Poland) supplemented with ampicillin (TOKU-E, Bellingham, Washington, USA, 100 μg/ml) at 37 °C with agitation until OD_600_ reached ~ 0.5 and then induced with 0.1 mM IPTG (isopropyl-β-D-thiogalactopyranoside (Bio-Connect, Huissen, The Netherlands)), followed by overnight incubation at 20 °C. Cultures were pelleted by centrifugation (5000 × *g*, 10 min, 4 °C) and resuspended in lysis buffer (0.5 M NaCl (Acros Organics, Thermo Fisher Scientific, Waltham, Massachusetts, USA), 20 mM NaH_2_PO_4_ (Acros Organics, Waltham, Massachusetts, USA), pH 7.4) and lysed by 3 cycles of freeze-thawing followed by sonication (15–30 s impulse, 30 s break, 3–5 times, power level 25%, on ice Bandelin Sonopuls HD 2070). The supernatant fraction was separated from cell debris by centrifugation (30 min, 12,500 × *g*, 4 °C) and filtered through 0.2 μm filter (Merck Millipore, Burlington, Massachusetts, USA) before application on the chromatographic column. For protein purification NGC Medium—Pressure Liquid Chromatography System (Bio-Rad, Hercules, California, USA) combined with Prepacked IMAC Nickel Columns (5 ml column volume, Bio-Rad, Hercules, California, USA) and long column wash (0.5 M NaCl, 20 mM NaH_2_PO_4_, pH 7.4), followed by a wash with buffer supplemented with 25 mM imidazole (Acros Organics, Waltham, Massachusetts, USA) to remove the unspecific binding. The protein was eluted with buffer supplemented with 500 mM imidasole and dialyzed against PBS (pH 7.4) overnight at 4 °C. Purified proteins were kept frozen at − 80 °C until use.

### Minimal Halo Forming (MHF) concentration measurement of depolymerase

The concentration of recombinant depolymerase was measured using the Qubit 2.0 fluorometer (Thermo Fisher Scientific, Waltham, Massachusetts, USA) according to the manufacturer protocol. The depolymerase was serially diluted in Tryptic Soy Broth (TSB, bioMerieux, Marcy l'Etoile, France) to following concentrations: 100 µg/ml, 10 µg/ml , 1 µg/ml and further in two-fold dilution series (500 ng/ml, 250 ng/ml, 125 ng/ml, 62.5 ng/ml, 31.25 ng/ml, 15.625 ng/ml, 7.8125 ng/ml and 3.90625 ng/ml). Ten-μl drops were spotted on the bacterial lawn to test the halo zone formation. As a control 10 μl of PBS and TSB was used (spot assay)^39,40^. Plates were incubated overnight at 37 °C and checked for the presence of visible opaque areas (halo zones). The MHF concentration was set as the lowest protein concentration at which a halo zone was still visible. The experiments were conducted in triplicate.

Halo forming ability of KP34p57 recombinant depolymerase was presented in Supplementary Materials as Figure S2. In this figure phages, KP34, and KP15 specificity test and depolymerase KP34p57 support for phage KP15 were presented. Figure S2a depicts a spot test on *K. pneumoniae* 77 bacterial lawn and S2b *K. pneumoniae* ATCC 700603 bacterial lawn.

### Minimal inhibitory concentration (MIC) determination of ciprofloxacin

The MIC of ciprofloxacin (MP Biomedicals, Thermo Fisher Scientific Waltham, Massachusetts, USA) was determined by the broth dilution method according to CLSI (Clinical Laboratory Standards Institute) recommendations. *Klebsiella pneumoniae* 77 grown on Mueller Hinton Agar (MHA, bioMerieux, Marcy l'Etoile, France) was resuspended in saline to 0.5 McFarland standard (approximately 1.5 × 10^8^ CFU/ml) using a densitometer (bioMerieux, Marcy l'Etoile, France). The serial dilutions of ciprofloxacin stock (5.1 mg/ml) were prepared in MHB according to CLSI recommendations and pipetted into 96 well plates (twofold dilution series in the range of 256 µg/ml–0.015 µg/ml). Bacteria were added to final CFU/ml reaching 5 × 10^5^ and incubated overnight at 37 °C. The absorbance (600 nm) was measured with Varioscan Lux (Thermo Fisher Scientific, Waltham, Massachusetts, USA). The MIC was established as the lowest concentration inhibiting the visual growth of microorganisms. The experiments were conducted in triplicate.

### Biofilm eradication assay

The overnight culture of *K. pneumoniae* 77 in TSB was centrifuged (5 min, 1500 × *g*). Pelleted cells were washed twice with saline (0.9% NaCl). 0.5 McFarland standard corresponding to 1.5 × 10^8^ CFU/ml was set in saline using a densitometer (bioMerieux, Marcy l'Etoile, France). The cells were further diluted in TSB and pipetted to 96 well plates. The polystyrene pegs were immersed in wells containing ~ 10^6^ CFU. Biofilm was formed in the humid chamber for 24 h, 48 h, and 72 h at 37 °C in static conditions. The lid with pegs was transferred to fresh TSB broth every 24 h. After biofilm formation, pegs were washed in saline and then transferred to fresh TSB containing different concentrations of depolymerase KP34p57 (1000 MHF, 100 MHF, 10 MHF, and 1 MHF), ciprofloxacin (MP Biomedicals, Thermo Fisher Scientific Waltham, Massachusetts, USA, 4 MIC, 2 MIC, 1 MIC, and 0.5 MIC) and phage KP34 (10^6^ and 10^9^ PFU/ml). The most optimal concentrations of antibiofilm compounds (10 MHF of depolymerase, 4 MIC of ciprofloxacin, and 10^8^ PFU/ml) were chosen for treatments in combinations. The different combinations were tested: depolymerase + antibiotic, depolymerase + phage, antibiotic + phage, depolymerase + phage + antibiotic against developed 72 h biofilm. Biofilm was treated with antimicrobial agents for 2 h at 37 °C. Then, pegs were washed twice in saline, and: (a) sonicated for 30 min in the ultrasonic cleaner with the addition of ice (160 W, Polsonic, SONIC-3, Warsaw, Poland) and serially diluted for colony count; (b) sonicated for 30 min in the ultrasonic cleaner with the addition of ice (160 W, Polsonic, SONIC-3, Warsaw, Poland) and stained with LIVE/DEAD BacLight Bacterial Viability Kit (Invitrogen, Thermo Fisher Scientific Waltham, Massachusetts, USA) [^41^, when used in microtiter assay^42^, when used with confocal laser scanning microscopy (CLSM)] in the F-bottom black 96-well plate according to the manufacturer protocol (the fluorescence intensity was measured at a wavelength centered at 530 nm with the excitation wavelength centered at 485 nm for live cells and a wavelength centered at 630 nm with the excitation wavelength centered at 485 nm for dead cells); (c) stained with 0.01% crystal violet solution for 15 min at room temperature and washed with saline for crystal violet assay. The dye was eluted from pegs via 15 min-long incubation in 96% ethanol and the absorbance was measured at 595 nm with Varioscan Lux (Thermo Fisher Scientific Waltham, Massachusetts, USA). Biofilm eradication experiments were conducted twice, each condition in five repeats.

### The statistical analysis

The statistical analysis was performed using the Brown-Forsythe ANOVA test and Welch’s ANOVA test (GraphPad Prism 8). The Dunnett's T3 test was run for multiple comparisons with adjusted *P*-value < 0.001 (indicated as two asterisks) and *P* values between 0.01 and 0.001 (indicated as one asterisk). Both *P* values were considered statistically significant.

### Protein visualization with SDS-PAGE

For protein visualization, sodium dodecyl sulfate–polyacrylamide gel electrophoresis (SDS-PAGE, 180 V for approximately 1 h) in 12% polyacrylamide gels was performed^43^. Protein samples were mixed with five times concentrated reducing sample buffer (Laemmli Sample buffer (Bio-Rad, Hercules, California, USA) supplemented with 2-mercaptoethanol, 5% v/v) in 1:4 ratio and boiled at 95 °C for 5 min. Precision Plus Protein Unstained ladder (Bio-Rad, Hercules, California, USA) was used for protein molecular weight (MW) estimation. For protein bands visualization Coomassie brilliant blue R-250 (Bio-Rad, Hercules, California, USA) staining was applied. SDS-PAGE micrograph, as a confirmation of successful overexpression and purification of recombinant depolymerase KP34p57, was added in Supplementary Materials as Figure S1a.

### Zymography analysis using extracted and purified exopolysaccharides

The extraction and purification of EPS/CPS were done as described by Bales et al.^44^. *Klebsiella pneumoniae* 77 biofilm was grown for 5 days at 37 °C in static conditions. The culture was started by adding 20 ml of overnight *K. pneumoniae* culture into a flask with a broad bottom and 200 ml TSB (to provide a large surface for biofilm formation). To every 10 ml of biofilm sludge 60 μl of formaldehyde was added (to prevent cell lysis) and incubated at RT with shaking for 1 h followed by the addition of 4 ml of 1 M NaOH per every 10 ml of sludge. During 3 h-incubation with shaking, the EPS/CPS was extracted. The supernatant containing EPS/CPS was separated by centrifugation (16,800 × *g*, 1 h, 4 °C) and dialyzed against distilled water (12–14 kDa molecular weight cut-off membrane, SERVA Electrophoresis GmbH, Heidelberg, Germany) overnight. Trichloroacetic acid (TCA, SERVA Electrophoresis Gmbh, Heidelberg, Germany) was added to the concentration 20% and incubated on ice for 0.5 h, for proteins and nucleic acids precipitation. To supernatant, separated by centrifugation (16,800 × *g*, 1 h, 4 °C), 1.5 volumes of cold 96% ethanol (VWR, Radnor, Pennsylvania, USA) was added to precipitate exopolysaccharides from lipids, 24 h at − 20 °C. EPS/CPS was pelleted by centrifugation (16,800 × *g*, 1 h, 4 °C), resuspended in ultrapure water, and dialyzed against Milli-Q (12–14 kDa molecular weight cut-off membrane, SERVA Electrophoresis Gmbh, Heidelberg, Germany) overnight. The EPS/CPS solution was lyophilized and resuspended in ultrapure water to desired concentration.

For zymography analysis standard SDS-PAGE gels containing EPS/CPS solution were prepared (5 mg/gel)^45^. The depolymerase sample was mixed with Laemmli sample buffer in the 1:1 ratio and denatured for 5 min at 95 °C. After SDS-PAGE under standard condition gels were washed three times in ultrapure water (10 min with agitation) followed by 48 h—long incubation in renaturation buffer (0.15 M NaH_2_PO_4_ (Acros Organics, Waltham, Massachusetts, USA), 0.010 M MgCl_2_ (VWR, Radnor, Pennsylvania, USA); pH 7.0, 0.1% (v/v) Triton X-100 (SERVA, Heidelberg, Germany)) with gentle agitation. Gels were again washed three times in ultrapure water (10 min with agitation), stained (methylene blue 0.1% (w/v), 0.001% (w/v) KOH (VWR, Radnor, Pennsylvania, USA)) for 12 h and destained in ultrapure water until the opaque bands were visible. Zymography image, as a confirmation of recombinant depolymerase KP34p57 specificity to extracted EPS/CPS from *K. pneumoniae* 77 culture was added in Supplementary Materials as Figure S1b.

### Influence of sonication on K. pneumoniae viability

*Klebsiella pneumoniae* 77 culture in TSB (bioMerieux, Marcy l'Etoile, France) was diluted to 0.5 McFarland standard (approximately 1.5 × 10^8^ CFU/ml) using a densitometer (bioMerieux, Marcy l'Etoile, France) and sonicated for 30 min in the ultrasonic cleaner with the addition of ice (160 W, Polsonic, SONIC-3, Warsaw, Poland) and serially diluted for colony count or kept on ice for 30 min and serially diluted for colony count, as a control. Dilution series were spotted on TSA (bioMerieux, Marcy l'Etoile, France) and after overnight incubation at 37 °C colonies were counted to calculate CFU. The experiment was conducted in four repeats. The comparison of CFU/ml after and without sonication procedure can be found in Supplementary Materials as Figure S3.

### Ciprofloxacin activity on planktonic cells verified by LIVE/DEAD BacLight Bacterial Viability Kit and fluorescence microscopy

*Klebsiella pneumoniae* 77 culture in TSB (bioMerieux, Marcy l'Etoile, France) was diluted to 0.5 McFarland standard, using a densitometer (bioMerieux, Marcy l'Etoile, France) and treated for 2 h with ciprofloxacin (MP Biomedicals, Thermo Fisher Scientific Waltham, Massachusetts, USA, a final concentration of 1 µg/ml, corresponding to 4 MIC for *K. pneumoniae* 77). After this time cultures were centrifuged (5 min, 1500 × *g*), pelleted cells were resuspended in the original volume of saline (0.9% NaCl) and stained with LIVE/DEAD BacLight Bacterial Viability Kit (Invitrogen, Thermo Fisher Scientific Waltham, Massachusetts, USA) according to the manufacturer protocol. Briefly, 6 µl of SYTO 9 was mixed with 6 µl of propidium iodide and added to 2 ml of filter-sterilized Gibco Distilled Water (Thermo Fisher Scientific Waltham, Massachusetts, USA) and used for 1:1 (v:v) dilutions with the bacterial suspensions. After 15 min incubation in the darkness, suspensions were five times concentrated via pelleting and resuspending in one-fifth volume of saline. Ten µl drop was used for visualization with Axio Imager M1 upright wide-field fluorescence microscope (Carl Zeiss, Germany; an illuminator Zeiss HBO 100; a 100 × oil immersion objective Zeiss Plan-Neofluar 100 ×/1.30). Exposure times were 50 ms. Filter sets were FITC (for SYTO 9) and Texas Red (for propidium iodide). Pictures were taken with Zeiss AxioCam MRC digital color camera combined with Zeiss AxioVision 4.5 software. Pictures taken as the confirmation that LIVE/DEAD BacLight Bacterial Viability Kit can be used for the differentiation between live and dead cells after ciprofloxacin treatment have been added in Supplementary Materials as Figure S4. The antibacterial activity of ciprofloxacin was also confirmed with the colony count.

## Supplementary information

Supplementary Information.
