# Supplementary material for: Advantages and limitations of microtiter biofilm assays in the model of antibiofilm activity of Klebsiella phage KP34 and its depolymerase
Source: Sci Rep. 2020 Nov 23;10:20338. doi: 10.1038/s41598-020-77198-5 (PMC7683578; doi:10.1038/s41598-020-77198-5)
Supplement: Supplementary file 1 — Supplementary Information. [file 41598_2020_77198_MOESM1_ESM.docx]

**Advantages and limitations of microtiter biofilm assays in the model of antibiofilm activity of *Klebsiella* phage KP34 and its depolymerase**

Agnieszka Latka**^1^** and Zuzanna Drulis-Kawa**^1^***

**^1^***Department of Pathogen Biology and Immunology, Institute of Genetics and Microbiology, University of Wroclaw, ul. S. Przybyszewskiego 63, 51-148 Wrocław, Poland.*

agnieszkalatka1989@gmail.com, zuzanna.drulis-kawa@uwr.edu.pl

Correspondence to: [zuzanna.drulis-kawa@uwr.edu.pl](mailto:zuzanna.drulis-kawa@uwr.edu.pl)

**Supplementary materials**

**a b**

MM D D

**
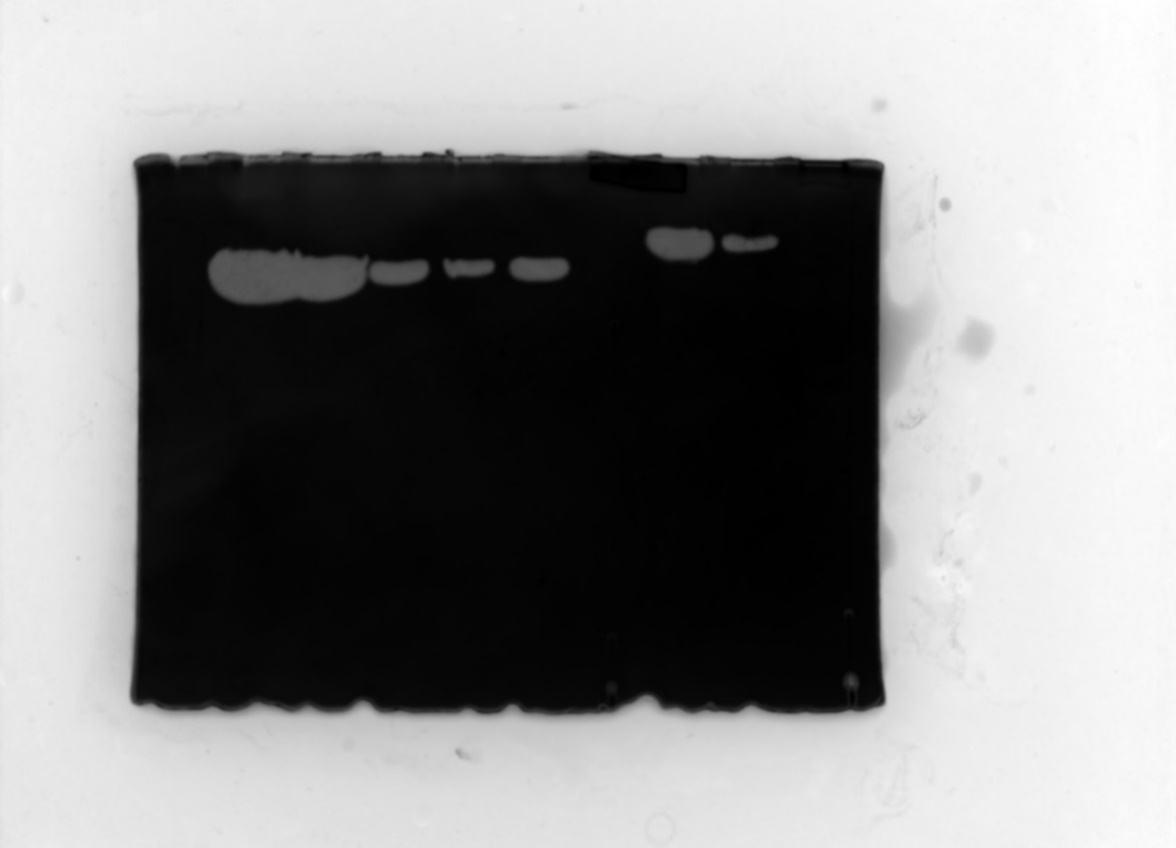

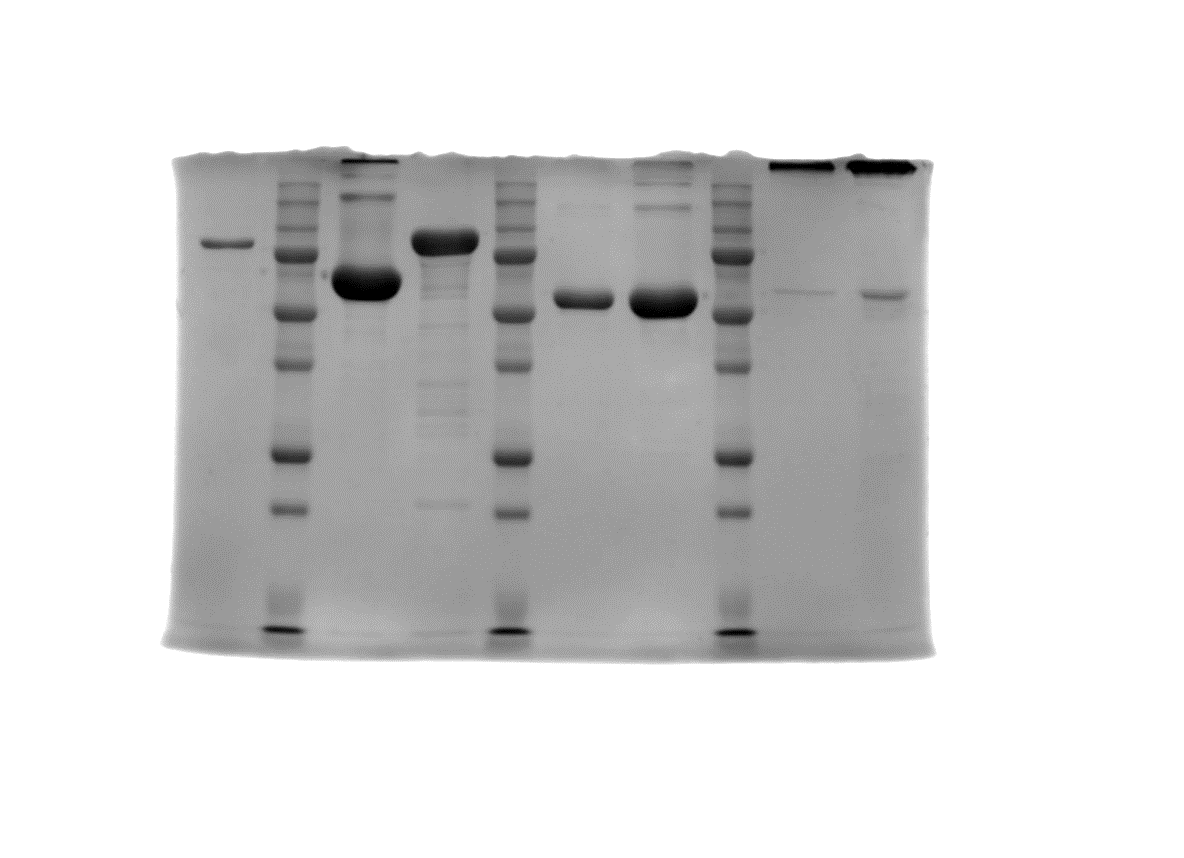
**

250

150

100

75

50

37

25

20

15

10

**Figure S1.** **Expression and zymography of recombinant depolymerase KP34p57**. Purified KP34p57 (a), the enzymatic activity of KP34p57 depolymerase against exopolysaccharides extracted from *K. pneumoniae* 77 culture proved by zymography analysis (b). With “D” marked lanes where the depolymerase was loaded and (in the case of zymography) polysaccharide degradation effect is visible. With “MM” marked lane where the mass marker was loaded (Precision Plus Protein Standards, Bio-Rad).


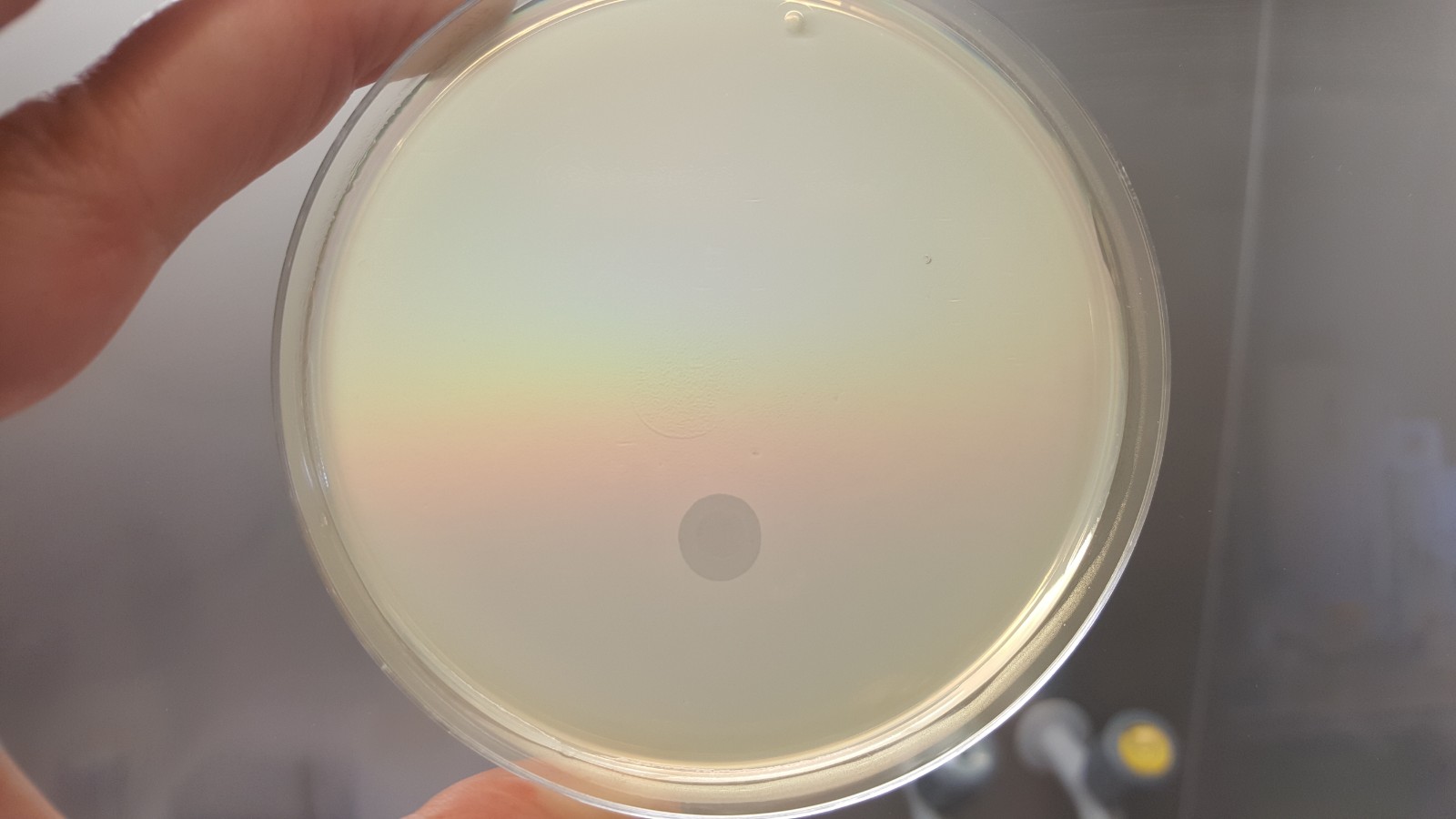


phage KP15

phage KP34 depo KP34p57

*K. pneumoniae* ATCC 700603


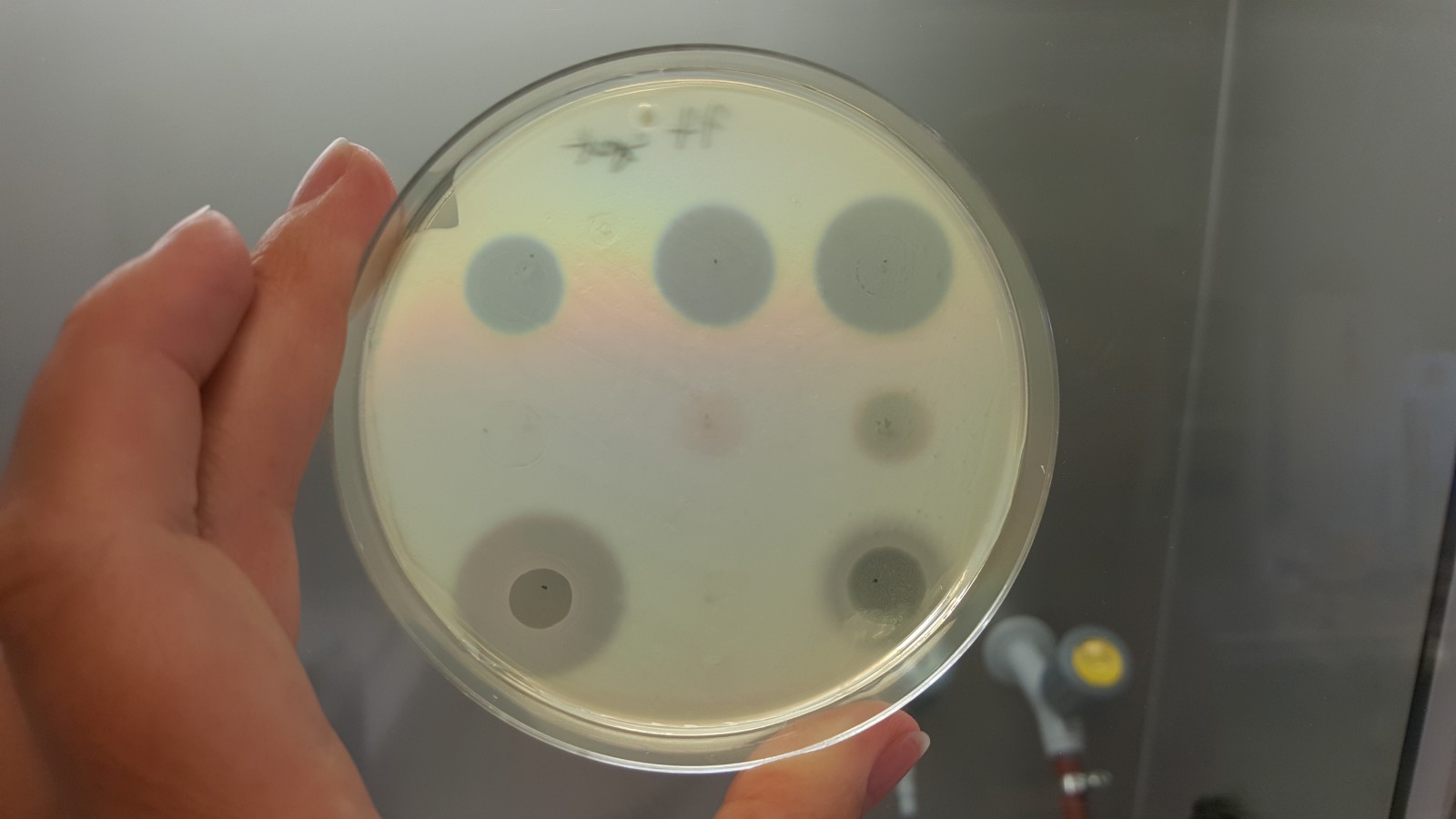


depo KP34p57

300 µg/ml 30 µg/ml 3 µg/ml

depo KP34p57

0.3 µg/ml 0.03 µg/ml 0.003 µg/ml

phage KP34 phage KP15

depo KP34p57

+ phage KP15

*K. pneumoniae* 77

phage KP15 phage KP34

depo KP34p57

+

phage KP15

0.003 µg/ml 0.03 µg/ml 0.3 µg/ml

**Figure S2. Spot test assay on the bacterial lawn to determine KP34 and KP15 phages host range and depolymerase KP34p57 specificity together with a minimal halo forming concentration (MHF).** Left panel: *K. pneumoniae* 77 is susceptible to phage KP34 infection and capsule degradation by KP34p57 recombinant enzyme (10-fold dilutions of depolymerase KP34p57), but not susceptible to phage KP15 infection. Phage KP15 can infect *K. pneumoniae* 77 only with the previous capsule degradation by depolymerase KP34p57. Right panel: *K. pneumoniae* ATCC 700603 is a host for phage KP15, but not for phage KP34. Depolymerase KP34p57 is not specific to *K. pnemoniae* ATTC 700603 capsular polysaccharides (no halo zone visible).


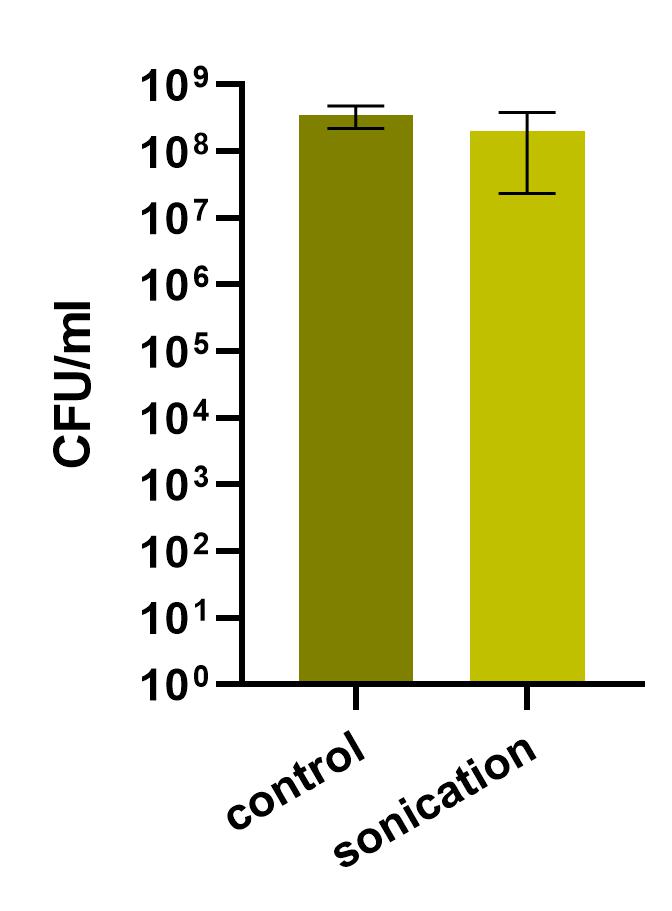


**Figure S3.** **Influence of sonication during 30 min using an ultrasonic cleaner on *K. pneumoniae* 77 colony count.** There were no statistically significant differences in CFU count of sonicated versus non-sonicated bacteria (*P*-value = 0.7072).


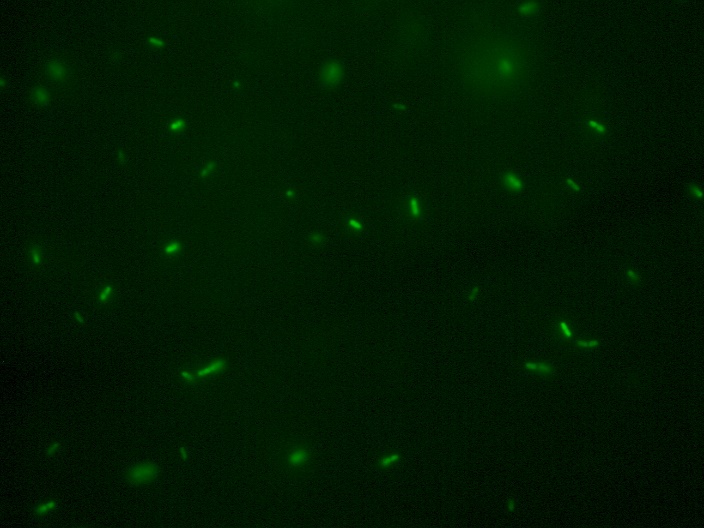

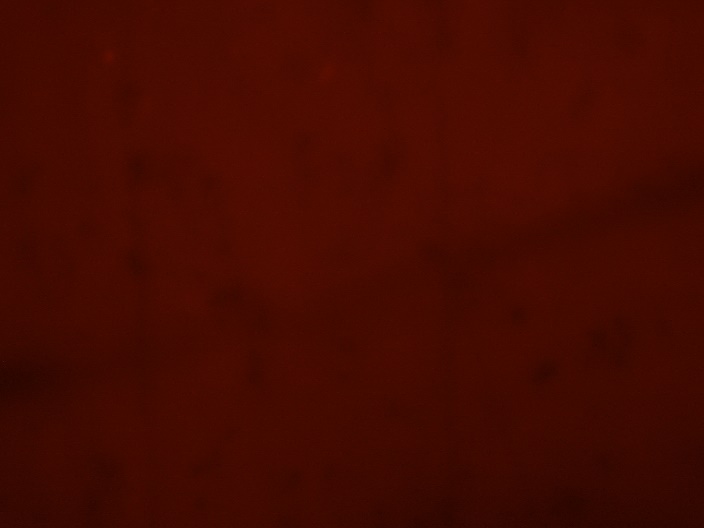

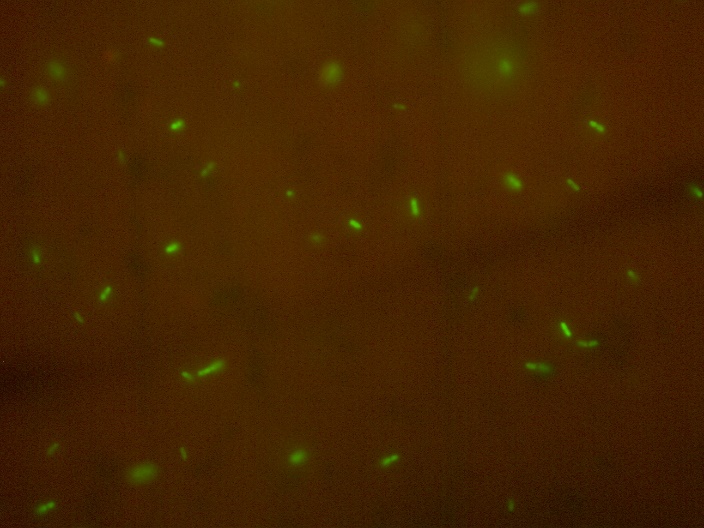


**d e f**

**a b c**


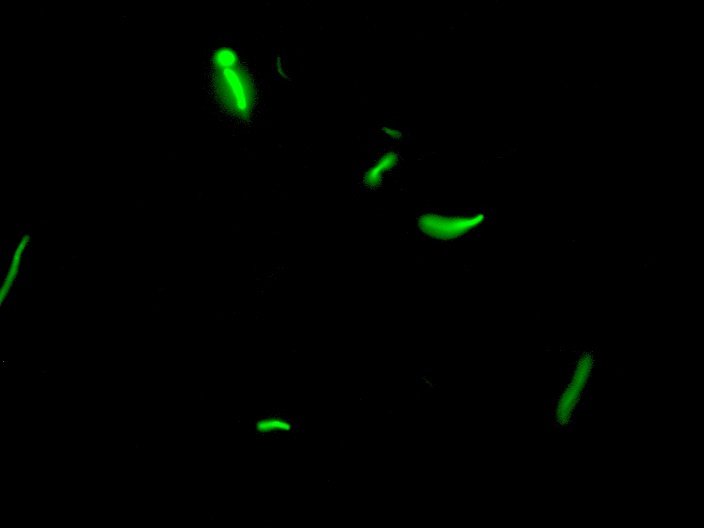

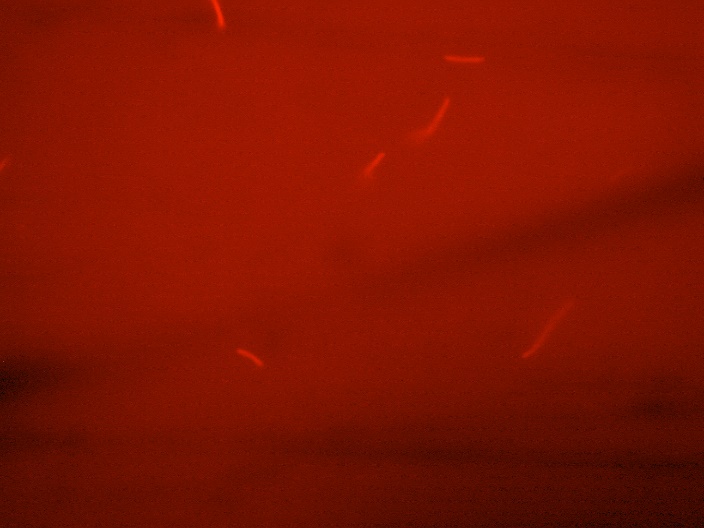

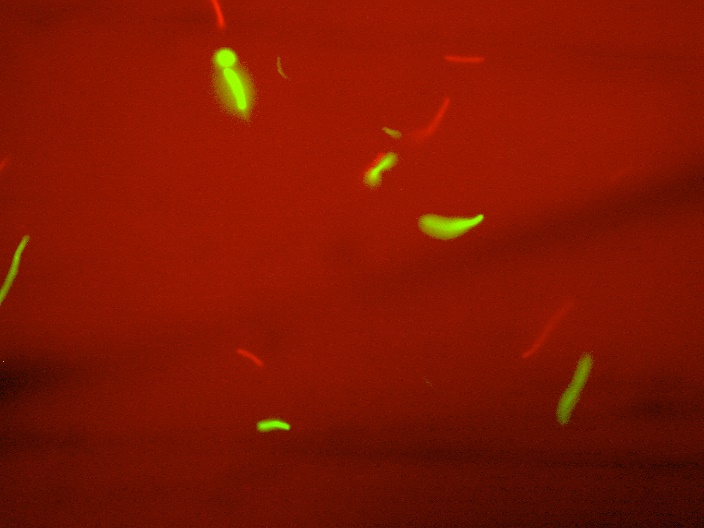


**Figure S4.** **The antibacterial activity of ciprofloxacin on the planktonic culture of *K. pneumoniae* 77 tested with LIVE/DEAD BacLight Bacterial Viability Kit and examined with fluorescent microscopy.** The visualization was done with Axio Imager M1 upright wide-field fluorescence microscope (Carl Zeiss, Germany; an illuminator Zeiss HBO 100; a 100 × oil immersion objective Zeiss Plan-Neofluar 100x/1.30); exposure times 50 ms; filter sets FITC for SYTO 9 and Texas Red for propidium iodide. Pictures were taken with Zeiss AxioCam MRc MRC digital color camera combined with Zeiss AxioVision 4.5 software. Bacterial cells not treated with antibiotic (control) (a-c). Cells treated with ciprofloxacin (1 µg/ml, concentration corresponding to 4 MIC) for 2 h (d-f). Micrographs (a) and (d) show live bacteria stained with SYTO 9 (FITC filter); (b) and (e) show bacteria stained with propidium iodide (Texas Red filter); (c) and (f) show the combined micrographs. In the control sample there are plenty of small live bacterial cells (a), while dead bacteria are not found (b). Due to ciprofloxacin treatment we could observe an decrease in cell count (d), and morphological changes in bacteria (elongated cells). Bacterial cells penetrated by propidium iodide and stained red (e) are classified as dead. The standard colony count showed the reduction of 2.5 logs in *K. pneumoniae* 77 CFU/ml in the treatment sample versus control.
